# Impact of patent ductus arteriosus shunt size and duration on risk of death or severe respiratory morbidity in preterm infants born in China

**DOI:** 10.1007/s00431-022-04549-x

**Published:** 2022-07-15

**Authors:** Yingping Deng, Haiyan Zhang, Zhuoyu Zhao, Juan Du, Ruimiao Bai, Patrick J. McNamara

**Affiliations:** 1 Division of Neonatology, Children’s Hospital of Fudan University, 399 Wanyuan Street, Minhang District, Shanghai, 201102 China; 2 Department of Neonatology, Kunshan Maternity and Children’s Health Care Hospital, 5 Qingyang Middle Road, Kunshan, Jiangsu, 215300 China; 3 National Center for Children’s Health, Neonatal Center, Beijing Children’s Hospital, Capital Medical University, No. 56 Nanlishi Road, Xi Cheng District, Beijing, 100045 China; 4 Division of Neonatology, Northwest Women’s and Children’s Hospital, No. 1616 Yanxiang Road, Yanta District, Xi’an, 710061 China; 5 Department of Pediatrics, University of Iowa, 200 Hawkins Dr, Iowa City, IA 52242 USA

**Keywords:** Patent ductus arteriosus, Bronchopulmonary dysplasia, Respiratory morbidity, Preterm infants

## Abstract

The purpose of this study is to assess whether duration and size of the arterial duct were associated with severe respiratory morbidity and mortality in preterm infants. All echocardiography evaluations for patent ductus arteriosus (PDA) in a cohort of preterm infants, born at a gestational age less than 28 weeks, from birth up to 36 weeks of postconceptional age or final ductal closure were reviewed. Ductal size was measured at the pulmonary end. PDA was classified as small (E1: ductal diameter (DD) ≤ 1.5 mm), moderate (E2: 1.5 mm < DD ≤ 2.5 mm), or large (E3) (DD > 2.5 mm). The primary outcome was adverse outcome defined by the composite outcome of bronchopulmonary dysplasia (BPD) or death. Infants in whom the primary outcome occurred were classified as “high-risk” whereas patients who did not satisfy this outcome were classified as “low-risk”. Intergroup comparison (high vs. low risk) was performed using univariate and multivariate analyses. A total of 135 infants, born between 2010 and 2020, were evaluated. The primary outcome was satisfied in 46 (34.1%) patients. The high-risk group was characterized by increased duration of exposure to PDA of any (E1/E2/E3) grade (44 vs. 25.5 days, *p* = .0004), moderate or large (E2/E3) PDA (30.5 vs. 11.5 days, *p* < .0001), moderate (E2) PDA (10.8 vs.6 days, *p* = 0.05), and large (E3) PDA (11.5 vs.0 days, *p* < .0001) compared with low-risk group. Lower gestational age, prolonged duration of mechanical ventilation, higher rate of inotrope use, pharmacological therapy, and PDA ligation were also associated with development of BPD or death (high-risk group). After adjusting for confounders, the rate of inotrope use [OR 2.688, 95% CI (1.011–7.142), *p* = 0.047], duration of large (E3) PDA [OR 1.060, 95% CI (1.005–1.118), *p* = 0.03], and mechanical ventilation [OR 1.130, 95% CI (1.064–1.200), *p* = 0.0001] were independently associated with the composite of BPD or death. Among infants who developed BPD, 27 were classified as grade I and 18 as grade II BPD, respectively. Infants with grade II BPD had prolonged MV (20.0 vs. 9.0 days, *p* = 0.024), prolonged exposure to PDA of any grade (55.8 vs. 36.0 days, *p* = 0.03), and prolonged exposure to large (E3) PDA compared with infants with grade I BPD.

*   Conclusion*: Prolonged exposure to a large PDA was associated with severe respiratory morbidity and mortality in preterm infants. The modulator role of early intervention, in the most pathologic shunts, on severe respiratory morbidity in preterm infants should be tested in well-designed clinical trials.

**Table:** 

**What is Known:** *• Current guidelines recommended against accelerating PDA closure of preterm infants within 2 weeks of life, with low certainty evidence indicating improved long-term outcomes.* *• Recent studies suggest that conservative approach regarding PDA management has detrimental effects on the respiratory outcomes in a subgroup population.*	
**What is New:** *• Persistent patency of significant PDA is associated with increased risk of BPD/death in extremely preterm infants.* *• Targeted intervention of PDA is beneficial for the at-risk preterm infants with increased PDA hemodynamic significance.*

**What is Known:**

*• Current guidelines recommended against accelerating PDA closure of preterm infants within 2 weeks of life, with low certainty evidence indicating improved long-term outcomes.*

*• Recent studies suggest that conservative approach regarding PDA management has detrimental effects on the respiratory outcomes in a subgroup population.*

**What is New:**

*• Persistent patency of significant PDA is associated with increased risk of BPD/death in extremely preterm infants.*

*• Targeted intervention of PDA is beneficial for the at-risk preterm infants with increased PDA hemodynamic significance.*

Patent ductus arteriosus (PDA) is associated with increased risk of mortality and morbidities in premature infants [1]. Although numerous clinical trials have been conducted for the past 2 decades, no strategy has favorably mitigated risk of these outcomes [1–5]. Even in the recent PDA-TOLERATE trial, early treatment did not reduce the risk of bronchopulmonary dysplasia (BPD) or death [6]. Expert commentators have concluded that current evidence is insufficient to justify any universal approach to PDA care [7]. These data, in addition to reports of higher rates of spontaneous PDA closure, have led to a paradigm shift toward conservative management [8, 9]. Upon further review, several limitations of published trials exist that need thoughtful consideration before positively embracing an opinion against PDA treatment [1–5]. These include the lack of standardization of the diagnosis of a hemodynamically significant PDA (hsPDA) in clinical trials, high rate of medical treatment in the control arm, relevance of trials prior to 2000 to contemporary neonatal practice, influence of physician equipoise on the enrollment process, and the fact that medical treatment is only efficacious in 50–60% of patients.

Importantly, there is increased recognition that mere patency of the PDA is a less effective discriminator of hemodynamic significance and “at-risk” state than comprehensive assessment of shunt magnitude and/or duration. El-Khuffash et al. demonstrated that PDA score reliably predicts risk of BPD or death in extremely preterm infants [10]. Clyman et al. showed that risk of BPD or death increased in mechanically ventilated infants after 7 days of exposure to a moderate-to-large PDA [11]. Finally, increased risk of BPD was noted in centers with a non-interventional approach to PDA management [12, 13]. These studies suggest that an “all or none” approach to PDA treatment is unlikely to be of benefit. Several studies highlight the merits of comprehensive echocardiography evaluation of hemodynamic significance; however, advanced imaging techniques are not routinely available in Chinese centers. In addition, recent data demonstrated that ductal diameter (DD) positively correlates with left ventricular output (LVO), isovolumic relaxation time (IVRT), retrograde flow, and other echocardiographic markers for hsPDA, and a threshold of 2.5 mm is good predictor of hsPDA [14]. We hypothesize that longer duration of exposure to a large PDA is associated with increased risk of adverse respiratory outcome in Chinese neonates.

## Methods

### Study design

This was an observational, retrospective study conducted at an outborn neonatal intensive care unit (NICU) at the Children’s Hospital of Fudan University. On average, approximately 130 very preterm infants were admitted annually, among whom 15% were extremely preterm infants [15].

### Patients

All preterm infants born at a gestational age (GA) less than 28 weeks, who were admitted to NICU between January 2010 and December 2020, were considered eligible for inclusion. Exclusion criteria were major malformations, congenital heart disease, congenital infections, early death (within 72 h of birth), or admission after 48 h of age. Infants with echocardiography scans that confirmed no evidence of PDA flow were excluded. Infants of parents who did not wish to pursue ICU care were also excluded. Although most of these infants were clinically stable, their parents refused medical treatment due to financial strain or other social factors [16, 17]; specifically, “parents didn’t wish ICU care” refers to a parental decision to withdraw all care because they were *unable to afford the significant cost* of neonatal intensive care for the extremely preterm infants.

### Data collection

Maternal, perinatal, and neonatal data were obtained on all enrolled infants. Antenatal and neonatal characteristics, including GA and birth weight at delivery, sex, twin or multiple gestation, mode of delivery, 5-min Apgar score, the use of antenatal steroids, intrauterine growth retardation, the presence of pre-eclampsia, pregnancy-induced hypertension, maternal diabetes, and premature rupture of membranes, were collected. Data regarding the following neonatal outcomes and morbidities were also recorded: respiratory distress syndrome (RDS), culture positive and negative sepsis, duration of invasive mechanical ventilation (MV), pneumothorax, thrombocytopenia, acute pulmonary hypertension, need for inotropic drugs, PDA pharmacological therapy, PDA ligation, intraventricular hemorrhage (IVH) grade III or IV based on the Papile criterion, necrotizing enterocolitis (NEC) as defined by ≥ stage 2 (based on the Bell criteria), receipt of treatment for retinopathy of prematurity, periventricular leukomalacia (PVL) as defined by periventricular white matter injury on MRI at 36 to 40 weeks postmenstrual age (PMA), length of hospital stay, and death before discharge [18, 19]. Diagnostic criteria for RDS were considered by the presence of clinical signs of respiratory distress (grunting, tachypnea, and intercostal retractions), requiring a respiratory support (supplemental oxygen requirement and/or non-invasive or invasive ventilation) and chest radiograph appearances (decreased lung air content, reticulogranular patterns, and air bronchograms), and exclusion of other causes of respiratory failure [20, 21]. Diagnosis of pulmonary hypertension was made by echocardiography evidence of supra-systemic pulmonary pressures (estimated by peak velocity of tricuspid regurgitation and pressure gradient calculation using modified Bernoulli equation), pure right-to-left or bidirectional shunt through a PDA, paradoxical interventricular septal motion at end-systole based on clinical signs of hypoxic respiratory failure, and a pre- to post-ductal saturation difference of > 10% on pulse oximetry [22]. Prolonged duration of MV was defined as need for assisted respiratory support greater than 14 days. BPD was classified according to the criteria proposed by Jensen et al.: no BPD, no support; grade I BPD, nasal cannula oxygen < 2 L/min; grade II BPD, nasal cannula oxygen > 2 L/min or non-invasive positive airway pressure; grade III, receipt of invasive MV [23]. Pulmonary hemorrhage (PH) was diagnosed as the presence of bloody fluid from the upper respiratory tract or the endotracheal tube with significant change in respiratory requirements and evidence of new pulmonary infiltrates in the chest X-ray.

### PDA evaluation

All infants were referred for echocardiography evaluation within the first 2 postnatal days to screen for PDA, as per institutional protocol for the extremely preterm infants or based on clinical concerns (oxygenation difficulty measured by oxygenation index, episodes of oxygen desaturation or apnea, escalation of respiratory support, feeding intolerance or “NEC-like” abdominal distension, oliguria or acute renal failure, hemodynamic instability requiring cardiotropic agents, and metabolic acidosis), presence of a moderate-to-large PDA, and need for treatment. No follow-up scan was requested if DD was less than 1 mm and infants were considered clinically stable. Comprehensive two-dimensional echocardiography examinations were performed by attending cardiologists using the GE Vivid E9 ultrasound machine (GE Medical Systems, Milwaukee, WI, USA) and a 12 MHz transducer. The PDA was identified from a high parasternal short-axis view. PDA diameter was measured as the narrowest point, usually at the pulmonary end. Only cases with exclusive left to right flow on pulse Doppler were considered eligible for enrollment. Sequential follow-up studies were requested by the attending neonatologists based on clinical significance of the PDA and shunt size. Trained echocardiographers (Y.D. and Y.G.), who were blinded to clinical outcomes, reviewed all echocardiograms performed from birth up to either 36 weeks PMA or final ductal closure. The value of DD was estimated to 1 decimal. PDA were classified as E1 (DD ≤ 1.5 mm, small PDA), E2 (DD 1.6–2.5 mm, moderate PDA), and E3 (DD > 2.5 mm, large PDA). The duration of ductal patency was calculated and expressed in days. As echocardiograms were not repeated daily, the interval between 2 scans were assigned partly (50%) to the grade detected by the previous scan and partly (50%) to the grade detected on the subsequent scan. For example, if a PDA E2 was detected on postnatal day 6, but no follow-up study was performed until postnatal day 9 when a PDA E3 was detected, day 7 was classified as PDA E2 and day 8 as PDA E3, respectively. The duration from birth to the time of first scan was allocated fully to the grade detected on the first scan. The cumulative number of days for each PDA stage was then calculated. If late re-opening occurs after the documented ductal closure, we did not calculate the elapsed time. If PDA persists beyond 36 weeks PMA, the exposure time post 36 weeks PMA was not calculated.

### Approach to PDA care

The decision to treat (oral ibuprofen or acetaminophen) was based on the following criteria:

(i) Echocardiography evidence of a large PDA with left to right shunt (DD > 2 mm)
(ii) Evidence of the clinical effects of pulmonary over-circulation and systemic hypoperfusion resultant from increased left to right ductal shunting, such as worsening ventilatory settings (more than 10% change in fraction of inspired oxygen (FiO_2_) or need for mechanical ventilation from non-invasive respiratory support), radiological evidence of cardiomegaly and/or pulmonary over-circulation, or resistant hypotension [24]

The eligible infants received a 3-day treatment course of oral ibuprofen of 10 mg/kg, 5 mg/kg, and 5 mg/kg. Infants who presented with feeding intolerance or oliguria were treated with oral acetaminophen with dose of 15 mg/kg at 6-h intervals for 5 days. Success of treatment was defined by a reduction in PDA diameter to less than 1.5 mm. The decision to perform PDA ligation was made in consultation with the cardiovascular surgical team for infants who failed two courses of medical therapy and were dependent on MV or non-invasive respiratory support (requiring non-invasive mean airway pressure greater than 8 cm H_2_O and FiO_2_ > 30%) with signs of end-organ hypoperfusion (e.g., inability to tolerate increases in enteral feeding volume) and echocardiography evidence of a hemodynamically significant duct (DD > 2 mm).

### Outcomes

The primary outcome was a composite of death or a diagnosis of BPD at 36 weeks PMA. Secondary outcomes included PDA characteristics, medical and surgical intervention for PDA, and diagnosis of BPD.

### Group allocation

Infants who developed BPD or died were characterized as *high-risk group* and surviving infants without BPD as *low-risk group*.

### Ethical considerations

This study was approved by the Research Ethics Board of Children’s Hospital of Fudan University on December 8, 2021 [REB# (2021) 475].

### Statistical analysis

Analyses were performed with MedCalc Software v 19.6.4 (MedCalc Software, Ostend, Belgio). Sample size (*n* = 145) was calculated based on achieving 80% power to detect 25% difference between groups. The estimated BPD incidence was 40% in extremely preterm infants [15]. Descriptive statistics were used to summarize antenatal risk factors, neonatal demographics, ductal characteristics, treatment strategies, and neonatal morbidities for the total population. Statistical significance was set at *p* < 0.05. Data are presented as mean ± standard deviation (SD) or median (interquartile range, IQR). A binary logistic regression model was developed to investigate the relationship between PDA characteristics and the composite primary outcome based on variables with *p* < 0.1 on univariate analysis. Comparative evaluation was performed between *high-risk* vs. *low-risk* groups. Categorical variables were analyzed using the *χ*^2^ or Fisher exact test as appropriate. Continuous variables were tested for normality and intergroup comparison performed using Student’s *t* test or the Mann–Whitney *U* test. Subgroup analyses between infants with grade I vs. II BPD were performed. Interobserver variability for DD measurements was tested using the Lin concordance correlation coefficient (CCC) on 80 echocardiograms by two experienced operators where 0 to 0.89 (slight to fair); 0.90 to 0.95 (moderate); and 0.96 to 0.99 (substantial) agreement.

## Results

### Demographic characteristics

**Fig. 1:**
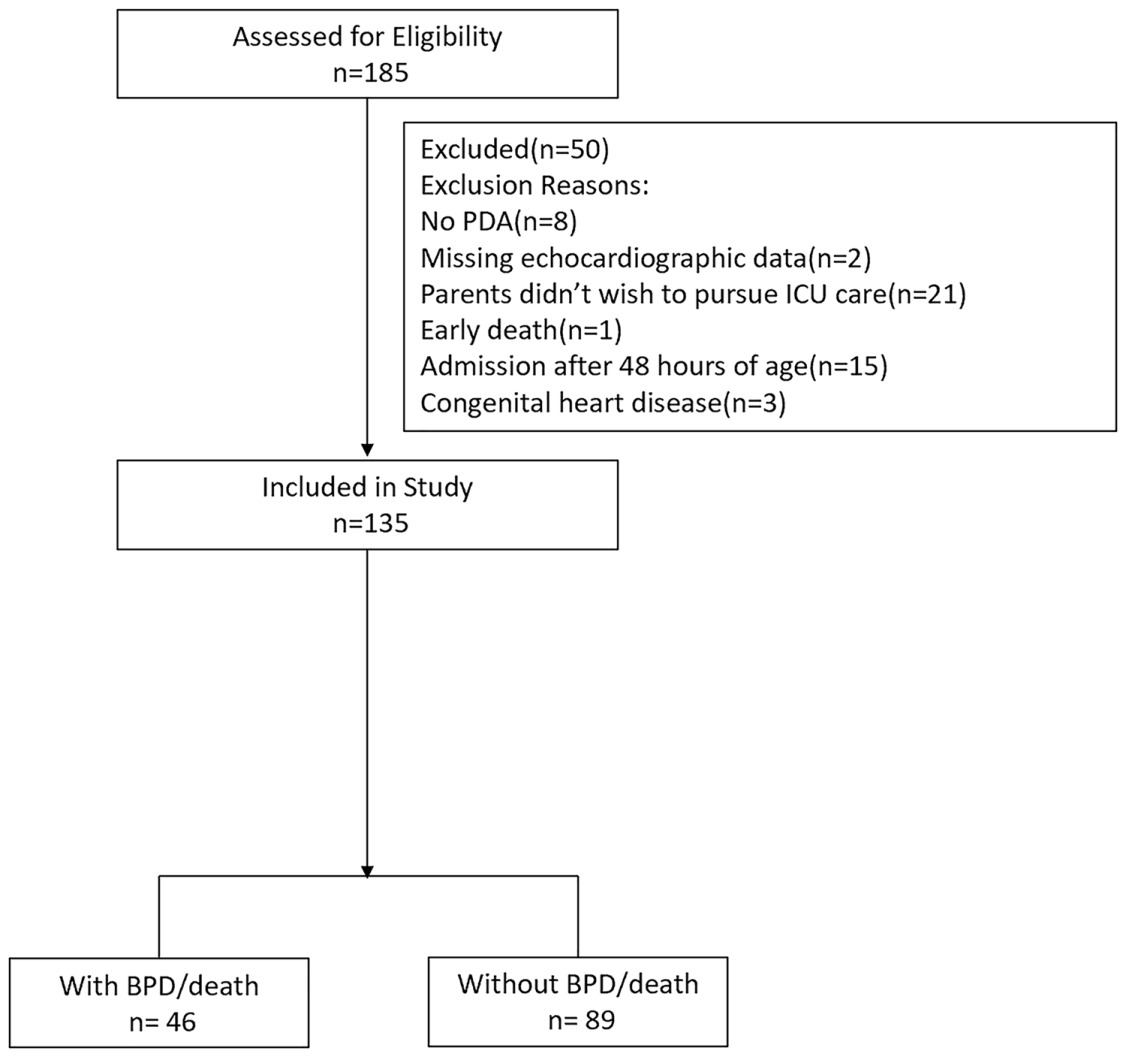
Diagram of included and excluded patients

A total of 185 preterm infants, born at a GA less than 28 weeks, were screened of whom 135 satisfied eligibility criteria (Fig. 1). The parents of 21 infants did not wish to pursue ICU care due to either perception of increased risk of adverse neurodevelopmental outcomes or financial hardship. For eligible patients, GA and weight at birth were 26.9 weeks (IQR 26.4–27.4 weeks) and 1009 ± 148.1 g respectively. In total, 46 (34.1%) infants developed BPD or died (*primary outcome*); of these, one infant died before 36 weeks PMA. Median duration of MV was 3.0 days (IQR 0–12.8 days). The study cohort was divided into high-risk (*n* = 46) and low-risk (*n* = 89) groups. There were no differences in baseline demographics between groups (Table 1).

**Table 1:** Clinical characteristics of preterm infants of high- and low-risk groups

	**High risk (*n* = 46)**	**Low risk (*n* = 89)**	***p* value**
GA (wk)	26.8 (24.1–27.9)	27.1 (24.9–27.9)	0.003**
Male, *n* (%)	26 (56.5%)	50 (56.2%)	0.97
Cesarean delivery, *n* (%)	36 (78.3%)	56 (62.9%)	0.07
Multiple gestation, *n* (%)	22 (47.8%)	38 (42.7%)	0.57
Antenatal steroids, *n* (%)	29/43	63/85	0.43
Pre-eclampsia, *n* (%)	1 (2.2%)	1 (1.1%)	0.99
PROM, *n* (%)	2/40	2/71	0.87
BW (g)	973.6 (± 137.4)	1027.6 (± 150.8)	0.5
Apgar at 5 min	8 (2–10)	8 (0–10)	0.55
RDS, *n* (%)	46 (100%)	86 (96.6%)	0.55
Surfactant treatment, *n* (%)	42 (91.3%)	71 (79.8%)	0.09
Duration of MV, d	12 (0–44)	2 (0–33)	< 0.0001**
Pneumothorax, *n* (%)	2 (4.3%)	1 (1.1%)	0.27
Pulmonary hemorrhage, *n* (%)	5 (10.9%)	2 (2.2%)	0.045*
Need for inotropic drugs, *n* (%)	25 (54.3%)	15 (16.9%)	< 0.0001**
Sepsis, *n* (%)	26 (56.5%)	44 (49.4%)	0.44
Pulmonary hypertension, *n* (%)	25 (54.3%)	35 (39.3%)	0.1
Length of hospitalization, d	99.5 (58–179)	74 (47–145)	< 0.0001**

^*^p < 0.05, ^**^p < 0.01; *d*, days

### PDA characteristics

A total of 515 echocardiograms were performed, with a median of 6 scans (IQR 3–8) per infant. The overall median postnatal age at first scan was 2 days (IQR 1–2). Median periods of E3 only, E2 + E3, or any grade (E1 + E2 + E3) PDA exposure were 4.0 days (IQR 0–11.9), 18.0 days (IQR 5.5–36.0), and 30.0 days (IQR 14.1–54.0) respectively. There were 10 infants who had echocardiography evidence of PDA and a small ductal shunt (DD ≤ 1 mm) on the first scan. In two of these infants, the PDA increased in size and required intervention at follow-up. Overall, 23 infants had persistent PDA at 36 weeks PMA. Interobserver CCC for DD was moderate [0.91 (95% CI 0.853–0.936)].

### PDA and outcome of BPD/death

Infants in the *high-risk* group (vs. low-risk group) were born at an earlier GA (26.8 vs. 27.1 weeks, *p* < 0.01), had a longer duration of MV (12 vs. 2 days, *p* < 0.0001), and had a higher incidence of inotrope use (54.3% vs. 16.9%, *p* < 0.0001). Infants in the high-risk group were more likely to have prolonged exposure to PDA of any grade (44 vs. 25 days, *p* = 0.0004), E2 or E3 (30 vs. 11 days, *p* < 0.0001), E2 only (11 vs. 6 days, *p* = 0.05), or E3 only (11 vs. 0 days, *p* < 0.0001) than low-risk infants (Table 2). High-risk infants were more likely to receive pharmacological therapy to achieve PDA closure (87.0% vs. 50.6%, *p* < 0.0001) and surgical ligation (30.4% vs. 5.6%, *p* = 0.0001). After adjustment for other risk factors on univariate analysis, inotrope use [OR 2.69, 95% CI (1.01–7.14), *p* = 0.047], duration of MV [OR 1.13, 95% CI (1.06–1.20), *p* = 0.0001], and duration of PDA E3 exposure [OR 1.060, 95% CI (1.01–1.12), *p* = 0.03] were independently associated with the development of BPD or death (Table 3).

**Table 2:** Relationship of ductal diameter and ductal patency and development of BPD or death

	**High-risk (*n* = 46)**	**Low-risk (*n* = 89)**	***p* value**
PDA any grade (E1/E2/E3), d	44 (5.5–79)	25.5 (1–72)	0.0004**
PDA grade E2/E3, d	30.5 (3–79)	11.5 (0–70)	< 0.0001**
PDA grade E2, d	10.8 (0–74.5)	6 (0–64.5)	0.05*
PDA grade E3, d	11.5 (0–64)	0 (0–33.5)	< 0.0001**
PDA ligation, *n* (%)	14 (30.4%)	5 (5.6%)	0.0001**
Pharmacological therapy, *n* (%)	40 (87.0%)	45 (50.6%)	< 0.0001**

^*^p < 0.05, ^**^p < 0.01; *d*, days

**Table 3:** Multivariate analysis of the composite outcome of death or BPD

	BPD/death	
	OR (95% CI)	*p*
GA (wk)	1.417 (0.672–2.987)	0.359
Duration on MV, d	1.130 (1.064–1.200)	0.0001**
Need for inotropic drugs	2.688 (1.011–7.142)	0.047*
PDA E3, d	1.060 (1.005–1.118)	0.033*
PDA E2E3, d	0.998 (0.959–1.039)	0.923
PDAE1/E2/E3, d	1.009 (0.977–1.043)	0.571

^*^p < 0.05, ^**^p < 0.01; *d*, days

### BPD severity and PDA shunt burden

Among the 45 infants who developed BPD, 27 were classified as Jensen grade I and 18 as Jensen grade II, respectively. Demographic and perinatal characteristics were comparable between groups. Infants with grade II (vs. grade I) had prolonged MV (20.0 vs. 9.0 days, *p* = 0.02), prolonged exposure to PDA of any grade (55.8 vs. 36.0 days, *p* = 0.03), and prolonged exposure to large (E3) PDA (20.0 vs. 9.0 days, *p* = 0.04) (Table 4).

**Table 4:** Correlation between ductal diameter and duration of ductal patency and severity of BPD

	**Grade 2 BPD (*n* = 18)**	**Grade 1 BPD (*n* = 27)**	***p* value**
PDA any grade (E1/E2/E3), d	55.8 (34.0–68.0)	36.0 (20.9–58.0)	0.031*
PDA grade E2/E3, d	34.5 (23.0–57.5)	25.0 (13.1–37.5)	0.147
PDA grade E2, d	7.3 (3.5–25.5)	12.5 (4.8–27.6)	0.424
PDA grade E3, d	20.0 (6.5–35)	9.0 (3.1–17.4)	0.046*
Duration of MV, d	20.0 (11.0–28.0)	9.0 (6.0–20.0)	0.024*
PDA ligation, *n* (%)	7 (38.9%)	7 (25.9%)	0.363
Pharmacological therapy, *n* (%)	16 (88.9%)	23 (85.2%)	1

^*^p < 0.05; *d*, days

## Discussion

Our data reaffirms the potential relationship of duration of exposure to PDA, MV, and risk of BPD. More importantly, in the absence of comprehensive echocardiography adjudication of PDA shunt volume, a larger PDA > 2.5 mm is an important threshold within Chinese NICUs to aid discrimination of patients at higher risk of death or BPD and triage suitability for medical intervention. Failure of normal postnatal closure of DA occurs in 40–55% preterm infants born less than 29 weeks’ gestation [25, 26]. Evidence from animal and human observational studies demonstrates that prolonged exposure to a hsPDA may result in pulmonary edema, worsening lung compliance, impaired pulmonary function, and arrested alveolar development which contribute to the development of BPD [27, 28]. Increased distance between the alveolar surface and the surrounding capillary bed limits the ease of gaseous molecular diffusion which clinically manifests as hypoxemia. These effects are mitigated by either increasing inspired oxygen and/or alveolar distending pressure. Preclinical data demonstrates, however, that exposure of the immature lung to hyperoxia leads to arrested alveolarization and the development of a heterogeneous, simplified pulmonary architecture [29]. In addition, prolonged MV exposure induces apoptosis of alveolar cells and inflammatory cascade in the neonatal lungs contributing to the development of BPD [30, 31].

In alignment with animal experimental evidence, data from the current study demonstrates that infants who developed BPD or died had a longer duration of MV, higher incidence of inotrope use, and prolonged exposure to PDA compared with infants in low-risk group. After adjustment for other risk factors, use of inotropes, duration of MV, and persistent exposure to a large PDA remained positively associated with BPD or death. Our results are consistent with findings from previous studies. El-Khuffash et al. demonstrated that a composite score of PDA severity based on the echocardiography criteria of hemodynamic significance measured at the time of PDA treatment predicts the later occurrence of BPD/death [10]. More recently, Schena et al. found that infants exposed to a more severe PDA, based on the McNamara and Sehgal PDA staging system, for a longer period are at increased risk of developing BPD. They found that with each additional week of exposure to an hsPDA, the risk of BPD is increased by 70% [32]. Finally, several observational studies have demonstrated that the association between PDA and BPD was apparent only when moderate-to-large shunts persist beyond 7–14 days [11, 33–35]. Our study provides additional evidence that longer duration of exposure to PDA diameter > 2.5 mm is an important determinant of mortality and risk of BPD. In addition, subgroup analysis showed that prolonged PDA exposure was also associated with severity of BPD.

Despite evidence of a strong associative relationship between the PDA and BPD, numerous randomized controlled trials of PDA treatment failed to demonstrate any consistent reduction in the rates of neonatal morbidities, including BPD [1–3, 5]. The side effects of medical treatment or surgical ligation have led to the trend toward conservative approach regarding PDA management. Lokku et al. demonstrated that, in Canadian centers, conservative PDA management increased, while pharmacotherapy and/or surgical ligation decreased between 2006 and 2012 [36]. Bixler et al. showed a significant decrease in diagnosis and medical/surgical treatment of PDA, with no evidence of increased morbidities, in a large cohort of premature infants from 280 NICUs across USA [37]. The discrepancy between the findings from epidemiologic studies and the results of randomized clinical trials may relate to the use of non-standardized definitions of hsPDA, variance in diagnostic criteria for BPD, and heterogeneity in clinical trial design. In addition, it is important to recognize that treatment was not uniformly effective in patients randomized to medical treatments, whereas patients in the control arm were not uniformly exposed to hsPDA due to higher-than-expected rates of spontaneous closure. It has been proposed that future trials on PDA treatment need to focus on high-risk premature infants with objective evidence of hemodynamic significance which will help to draw inference on the effect of PDA on clinical outcomes. In addition, it is crucial to perform a standardized assessment of the PDA in order to define the physiological variability or the magnitude of ductal shunt when selecting target population [38, 39].

The application of conservative therapy in both routine clinical practice and randomized clinical trials has been applied in a non-consistent manner. Although originally proposed as the use of shunt modulation strategies (based on the Hagen-Poiseulle principle) and/or fluid restriction or diuretics, its application includes an approach based on non-evaluation or non-consideration of the PDA. The safety of conservative treatment has not been formally evaluated until recently. *First*, Altit et al. reported that policy change to a strict non-intervention approach to PDA resulted in a 31% increase in the incidence of death/BPD among infants less than 26 weeks GA, whereas there was no change in outcomes among infants born between 26 and 29 weeks [12]. *Second*, Relangi et al. evaluated the impact of a less aggressive approach to PDA management on the development of BPD in premature infants. Fewer infants received PDA treatment (54% vs. 90%, *p* < 0.001), which was administered at a later age (9.8 vs. 5.6 days, *p* < 0.001) in epoch 2 (2011–2015), compared with a prior epoch (2005–2007). With a more conservative approach, infants in epoch 2 had greater odds of BPD, composite of BPD or death, and were more likely to receive treatment with postnatal steroids than those in epoch 1 [13].

The question of whether early targeted intervention of hsPDA positively mitigates neonatal morbidity has been the subject of recent consideration. Reliable determination of the modulator effect of PDA closure requires the conductance of well-designed clinical trials in which enrollment is restricted to patients at greatest risk of abnormal outcome, and where spontaneous closure is less likely thereby ensuring exposure of sufficient magnitude and duration, and treatment efficacy is guaranteed in the comparator group. The hemodynamic effects of the transductal shunt are influenced by factors including transductal diameter, balance between pulmonary and systemic vascular resistance, and the compensatory ability of the immature myocardium. Our imaging protocol was based on a limited appraisal of shunt volume, which may influence the reliability of our findings. Comprehensive echocardiography protocols, based on multimodal echocardiography parameters related to cardiac volume overload, systemic and/or end-organ perfusion, and myocardial performance in infants with PDA, may offer additional value in distinguishing a subpopulation of patients at increased risk of PDA-attributable morbidity. Several PDA scoring systems have been associated with BPD [10, 29, 40]. Increased access to neonatologists with the skill of echocardiography increases the feasibility of conducting clinical trials based on populations at risk.

The present study has several limitations, most importantly the retrospective nature of the data collection. In addition, the enrolled population was drawn from a single tertiary center which may have introduced selection bias. It is therefore uncertain whether our findings are broadly generalizable. Some infants were excluded because parents did not wish to pursue ICU care, due to personal financial or ethical concerns, which is a unique bias of the Chinese context. This is an important consideration and provides a putative explanation for the lower mortality rate in our cohort. In addition, it is possible that the unavailable or missing data in our study (status of clinical or histological chorioamnionitis, the use of prenatal dexamethasone, the grading of severity of RDS, and neonatal critical illness score) may lead to hidden bias due to unmeasured variables. Furthermore, we were unable to acquire details of the age at onset, number of episodes or status of blood culture of patients with sepsis. The competing effects of infection and inflammation in the setting of a PDA are important determinants of the risk of BPD. Lastly, to recruit a sufficient sample size, we collected data on patients over a long timeframe which may have introduced historical bias. Even though we examined the effects of multiple confounders, unmeasured differences in practice may also have influenced the rates of BPD/death and BPD. A large population-based multicenter study designed to assess the association between hsPDA and outcomes in preterm infants would be ideal to better answer this question. On the contrary, the strength of our study, which covered a wide time span, lies in the uniform and standard protocol of structural echocardiography with accurate measurement of ductal size and close monitoring based on patient’s medical conditions.

## Conclusion

Prolonged exposure to a large PDA is an important contributor to both mortality and BPD. Comprehensive appraisal of hemodynamic significance is an important consideration in determining the relative contribution of shunt volume to abnormal parenchymal versus vascular maldevelopment.

## Data Availability

Institutional Repository.

## Abbreviations

BPD: Bronchopulmonary dysplasia
CI: Confidence interval
DD: Ductal diameter
HsPDA: Hemodynamic significant PDA
IQR: Interquartile range
IVH: Intraventricular hemorrhage
MV: Mechanical ventilation
NICU: Neonatal intensive unit
OR: Odds ratio
PDA: Patent ductus arteriosus
PH: Pulmonary hemorrhage
PMA: Postmenstrual age
PVL: Periventricular leukomalacia
RDS: Respiratory distress syndrome
SD: Standard deviation
